# Supplementary material for: Genomic diversity of Escherichia coli from healthy children in rural Gambia
Source: PeerJ. 2021 Jan 6;9:e10572. doi: 10.7717/peerj.10572 (PMC7796664; doi:10.7717/peerj.10572)
Supplement: Supplemental Information 3 [file peerj-09-10572-s003.docx]

Supplementary File 3: Characteristics of the study population

| Sample ID | Lab ID | Age (months) | Gender | Bristol stool index | Domestic animal within household | Enrolment date |
| --- | --- | --- | --- | --- | --- | --- |
| 102135 | H1 | 43 | Female | Thick liquid | Goat, sheep | 18-Feb-09 |
| 102650 | H2 | 45 | Female | Soft | Goat, sheep, donkey | 27-Jul-09 |
| 103296 | H3 | 44 | Male | Soft | Goat, horse, donkey, rodent | 27-Apr-10 |
| 103298 | H4 | 44 | Male | Formed | Sheep, fowl, horse, donkey, rodent | 27-Apr-10 |
| 103621 | H5 | 37 | Female | Soft | Sheep, fowl, rodent | 01-Sep-10 |
| 103650 | H6 | 48 | Female | Soft | Fowl, donkey, rodent | 29-Sep-10 |
| 103649 | H7 | 45 | Female | Soft | Goat, sheep, fowl, horse, rodent | 29-Sep-10 |
| 103071 | H8 | 53 | Male | Formed | Goat, sheep, fowl | 15-Jan-10 |
| 103622 | H9 | 39 | Female | Soft | Goat, sheep | 01-Sep-10 |
| 100167 | H10 | 40 | Female | Soft | Goat, sheep, fowl | 01-Feb-08 |
| 100217 | H11 | 57 | Male | Formed | Cat, fowl, horse, rodent | 21-Feb-08 |
| 100230 | H12 | 51 | Male | Soft | Goat, sheep, cat, fowl, rodent | 28-Feb-08 |
| 100612 | H13 | 55 | Female | Formed | Goat, sheep, dog, fowl, horse, donkey, rodent | 16-Aug-08 |
| 100162 | H14 | 47 | Female | Thick liquid | Sheep, horse, donkey, rodent | 30-Jan-08 |
| 102255 | H15 | 42 | Male | Formed | Goat, sheep, fowl, horse, donkey, rodent | 26-Mar-09 |
| 102250 | H16 | 39 | Male | Formed | Fowl | 25-Mar-09 |
| 102114 | H17 | 54 | Male | Formed | Rodent | 12-Feb-09 |
| 102123 | H18 | 37 | Female | Soft | Goat, sheep, fowl, rodent | 14-Feb-09 |
| 103282 | H19 | 43 | Male | Formed | Goat, sheep, dog, cat, cow, fowl, | 22-Apr-10 |
| 100817 | H20 | 44 | Male | Soft | Dog, fowl | 03-Dec-08 |
| 100816 | H21 | 40 | Male | Soft | Goat, sheep, cow, fowl, horse, donkey, rodent | 03-Dec-08 |
| 102836 | H22 | 47 | Male | Thick liquid | Fowl, rodent | 12-Oct-09 |
| 102837 | H23 | 41 | Male | Thick liquid | Sheep, fowl, rodent | 12-Oct-09 |
| 102843 | H24 | 44 | Male | Soft | Fowl, rodent | 13-Oct-09 |
| 102907 | H25 | 36 | Male | Soft | Goat, sheep, fowl | 05-Nov-09 |
| 102905 | H26 | 37 | Male | Soft | Goat, sheep, fowl | 05-Nov-09 |
| 102262 | H27 | 38 | Male | Formed | Goat, sheep, rodent | 01-Apr-09 |
| 102728 | H28 | 41 | Male | Soft | Goat, fowl | 24-Aug-09 |
| 102729 | H29 | 41 | Male | Soft | Goat, dog, cat, fowl, donkey | 24-Aug-09 |
| 100806 | H30 | 55 | Male | Soft | Goat, sheep, dog, fowl | 21-Nov-08 |
| 102053 | H31 | 37 | Female | Formed | Cow, fowl, donkey, rodent | 29-Jan-09 |
| 102052 | H32 | 38 | Female | Formed | Goat, sheep, cow, fowl, donkey, rodent | 29-Jan-09 |
| 102511 | H33 | 37 | Male | Soft | Fowl, horse, donkey, rodent | 19-Jun-09 |
| 102649 | H34 | 37 | Male | Soft | Fowl, horse, donkey, rodent | 27-Jul-09 |
| 102454 | H35 | 52 | Male | Soft | Sheep, fowl, donkey, rodent | 02-Jun-09 |
| 102459 | H36 | 51 | Male | Formed | Goat, sheep, dog, cat, cow, horse, donkey, rodent | 04-Jun-09 |
| 100303 | H37 | 58 | Male | Formed | Sheep, fowl | 08-Apr-08 |
| 100320 | H38 | 42 | Female | Formed | Sheep, fowl, rodent | 19-Apr-08 |
| 100319 | H39 | 45 | Female | Formed | Goat, sheep, fowl, rodent | 17-Apr-08 |
| 103081 | H40 | 39 | Female | Thick liquid | Goat, sheep, fowl, horse, donkey, rodent | 20-Jan-10 |
| 103082 | H41 | 39 | Female | Thick liquid | Goat, sheep, fowl, horse, donkey, rodent | 20-Jan-10 |
| 100663 | H42 | 36 | Male | Thick liquid | Goat, sheep, fowl, donkey | 10-Sep-08 |
| 100072 | H43 | 51 | Female | Formed | Goat, cow, fowl, rodent | 03-Jan-08 |
| 103171 | H44 | 36 | Female | Soft | Goat, sheep, rodent, fowl, rodent | 18-Feb-10 |
| 103172 | H45 | 36 | Female | Soft | Goat, sheep, fowl, rodent | 18-Feb-10 |
| 103292 | H46 | 39 | Male | Soft | Goat, sheep, fowl | 23-Apr-10 |
| 102952 | H47 | 36 | Male | Soft | Goat, sheep, fowl, rodent | 20-Nov-09 |
| 102953 | H48 | 37 | Male | Soft | Goat, sheep, fowl, rodent | 20-Nov-09 |
| 102964 | H49 | 40 | Female | Formed | Goat, fowl, rodent | 26-Nov-09 |
| 102966 | H50 | 37 | Female | Formed | Goat, sheep, fowl, horse, donkey, rodent | 22-Apr-10 |
| 103281 | H51 | 44 | Male | Formed | Goat, sheep, dog, cat, fowl | 22-Apr-10 |
| 100540 | H52 | 43 | Male | Soft | Goat, sheep, fowl, rodent | 22-Jul-08 |
| 103123 | H53 | 38 | Male | Soft | Sheep | 03-Feb-10 |
| 103124 | H54 | 36 | Male | Soft | Fowl | 03-Feb-10 |
| 102089 | H55 | 38 | Female | Soft | Goat, cow, fowl, horse, donkey, rodent | 05-Feb-09 |
| 103297 | H56 | 38 | Male | Soft | Goat, sheep, fowl, horse, donkey, rodent | 27-Apr-10 |
| 102251 | H57 | 39 | Male | Formed | Fowl | 25-Mar-09 |
| 103602 | H58 | 38 | Female | Formed | Goat, sheep, cow, fowl | 26-Aug-10 |
| 103600 | H59 | 39 | Female | Formed | Goat, sheep, fowl | 26-Aug-10 |
| 100026 | H60 | 49 | Female | Soft | Goat, sheep, cow, fowl | 14-Dec-07 |
| 102102 | H61 | 47 | Female | Opaque watery | None | 11-Feb-09 |
| 102263 | H62 | 38 | Male | Formed | Horse, donkey, rodent | 01-Apr-09 |
| 103070 | H63 | 58 | Male | Soft | Goat, sheep, fowl | 15-Jan-10 |
| 103130 | H64 | 40 | Male | Soft | Sheep, fowl | 03-Feb-10 |
| 102051 | H65 | 36 | Female | Formed | Goat, sheep, dog, cat, cow, fowl, donkey, rodent | 29-Jan-09 |
| 102524 | H66 | 36 | Male | Soft | Goat, sheep, fowl, horse, donkey, rodent | 24-Jun-09 |
