# Supplementary material for: Genomic diversity of Escherichia coli from healthy children in rural Gambia
Source: PeerJ. 2021 Jan 6;9:e10572. doi: 10.7717/peerj.10572 (PMC7796664; doi:10.7717/peerj.10572)
Supplement: Supplemental Information 7 [file peerj-09-10572-s007.docx]

Supplementary File 7: Closest relatives to the study isolates

| Sample ID | 7-gene ST | Neighbour host | Neighbour status | Neighbour’s country of isolation | Allelic distance |
| --- | --- | --- | --- | --- | --- |
| H-32_5 | 10 | Human | Unknown | UK | 18 |
| H-36_1 | 59 | Human | Unknown | UK | 18 |
| H-39_1 | 452 | Human | Commensal | UK | 26 |
| H-9_1 | 2705 | Livestock |  | China | 29 |
| H-18_3 | 9274 | Human | Commensal | Unknown | 34 |
| H-2_1 | 9274 | Human | Commensal | Unknown | 34 |
| H-22_1 | 316 | Human | Commensal | UK | 35 |
| H-38_1 | 394 | Human | Pathogen (cystitis) | US | 39 |
| H-25_4 | 337 | Human | Unknown | Mali | 43 |
| H-37_1 | 5148 | Human | Pathogen (diarrhoea) | Ecuador | 43 |
| H-26_1 | 641 | Livestock |  | US | 46 |
| H-26_5 | 398 | Poultry |  | Kenya | 47 |
| H-48_2 | 485 | Human | Commensal | Tanzania | 57 |
| H-15_1 | 9277 | Human | Commensal | Zambia | 68 |
| H-15_2 | 747 | Human | Commensal | Egypt | 72 |
| H-28_1 | 469 | Human | Commensal | Kenya | 77 |
| H-21_2 | 1204 | Avian |  | Kenya | 81 |
| H-34_2 | 10 | Livestock |  | UK | 83 |
| H-38_2 | 58 | Human | Pathogen (bloodstream infection) | Australia | 87 |
| H-34_4 | 1727 | Unknown | Unknown | Unknown | 89 |
| H-35_1 | 226 | Human | Commensal | China | 93 |
| H-21_1 | 58 | Unknown | Unknown | Unknown | 98 |
| H-21_4 | 540 | Human | Unknown | Belgium | 100 |
| H-32_2 | 2175 | Livestock |  | UK | 100 |
| H-26_2 | 10 | Livestock |  | US | 111 |
| H-32_1 | 101 | Unknown | Unknown | Unknown | 111 |
| H-50_2 | 515 | Environment |  | Canada | 117 |
| H-41_1 | 43 | Unknown | Unknown | Unknown | 120 |
| H-26_4 | 2741 | Human | Commensal | Germany | 126 |
| H-50_1 | 410 | Livestock |  | US | 140 |
| H-18_1 | 38 | Poultry |  | US | 144 |
| H-21_5 | 223 | Unknown | Unknown | Unknown | 145 |
| H-40_1 | 155 | Unknown | Unknown | US | 146 |
| H-41_2 | 9283 | Environment | Commensal | US | 191 |
| H-36_4 | 9278 | Avian |  | Kenya | 208 |
| H-9_3 | 2914 | Canine |  | UK | 272 |
| H-9_5 | 29 | Unknown | Unknown | Unknown | 288 |
| H-34_1 | 603 | Laboratory |  | UK | 325 |
| H-55_1 | 9279 | Environment |  | Unknown | 333 |
| H-18_2 | 9281 | Unknown | Unknown | France | 430 |
| H-25_1 | 181 | Human | Commensal | Tanzania | 607 |
