# Supplementary figures and images for: Genomic diversity of Escherichia coli from healthy children in rural Gambia
Source: PeerJ. 2021 Jan 6;9:e10572. doi: 10.7717/peerj.10572 (PMC7796664; doi:10.7717/peerj.10572)

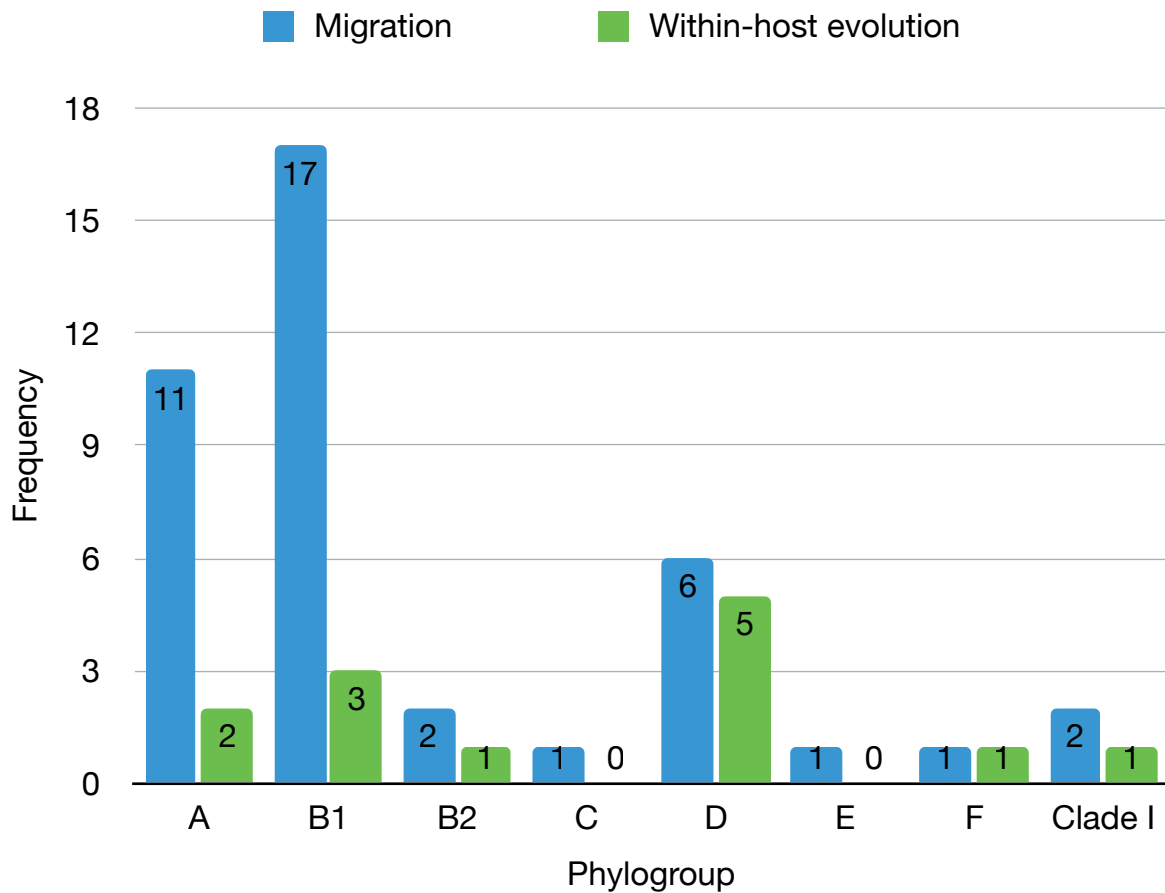

Supplement: Supplemental Information 10 — The distribution of variants inferred to have arisen from immigration events compared to those generated by within-host evolution by phylogroup [file peerj-09-10572-s010.pdf]

# ST(Achtman 7 Gene MLST)

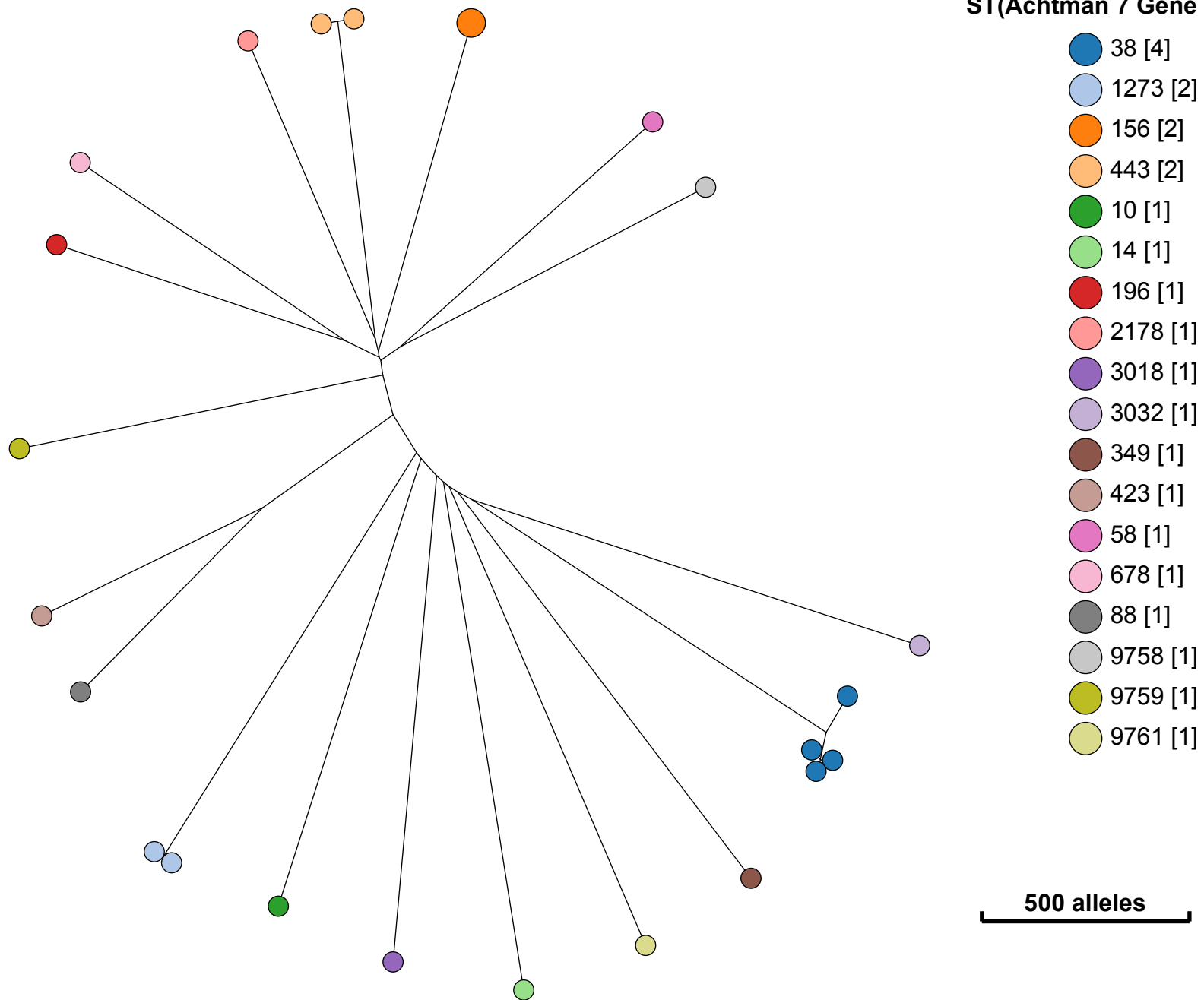

Supplement: Supplemental Information 11 — The Sequence types identified in these isolates are shown in the legend, with the genome count displayed in square brackets next to the respective sequence types. Three STs (ST38, ST58 and ST10) overlapped with what was found among commensal strains from this study (see Fig. 2). [file peerj-09-10572-s011.pdf]

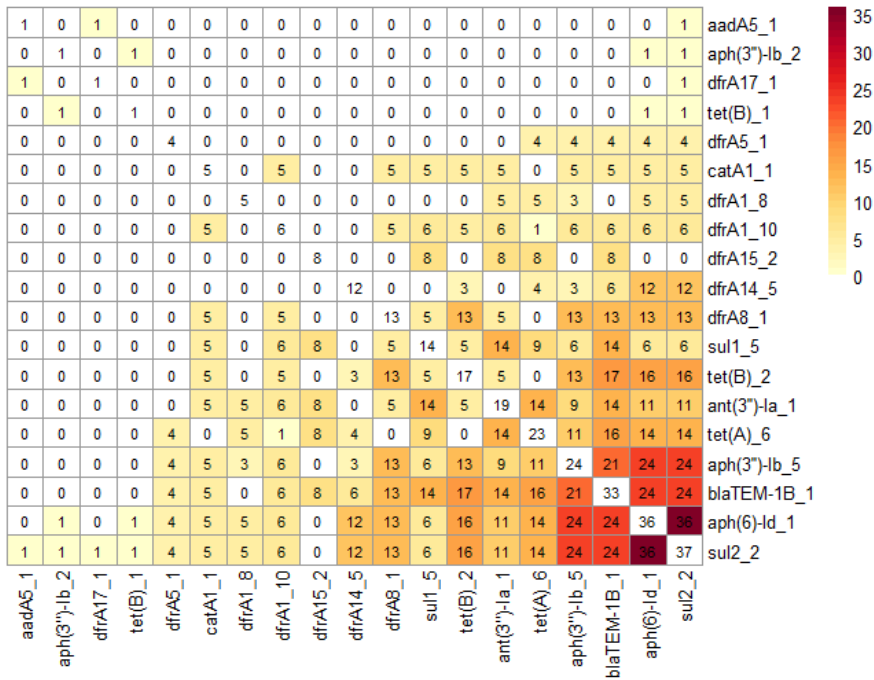

Supplement: Supplemental Information 12 — The diagonal values show how many isolates each individual gene was found in, while the intersections between the columns represent the number of isolates in which the corresponding antimicrobial resistance genes co-occurred [file peerj-09-10572-s012.pdf]
